# Dynamics of transmission of *Plasmodium falciparum *by *Anopheles arabiensis *and the molecular forms M and S of *Anopheles gambiae *in Dielmo, Senegal

**DOI:** 10.1186/1475-2875-7-136

**Published:** 2008-07-23

**Authors:** Mamadou Ousmane Ndiath, Cécile Brengues, Lassana Konate, Cheikh Sokhna, Christian Boudin, Jean François Trape, Didier Fontenille

**Affiliations:** 1 Institut de Recherche pour le Développement, Laboratoire de Paludologie et de Zoologie médicale, IRD Hann, BP 1386, Dakar, Sénégal; 2 Institut de Recherche pour le Développement, UR016, B.P. 64501, 34397, Montpellier, France; 3 Université Cheikh Anta Diop de Dakar, BP 5005, Dakar, Sénégal

## Abstract

**Background:**

The adaptation of *Anopheles gambiae *to humans and its environment involves an ongoing speciation process that can be best demonstrated by the existence of various chromosomal forms adapted to different environments and of two molecular forms known as incipient taxonomic units.

**Methods:**

The aim of this study was to compare the epidemiologic role of *Anopheles arabiens *is and the molecular forms M and S of *Anopheles gambiae *in the transmission of Plasmodium in a rural areas of southern Senegal, Dielmo. The sampling of mosquitoes was carried out monthly between July and December 2004, during the rainy season, by human volunteers and pyrethrum spray catches.

**Results:**

*Anopheles arabiensis*, *An. gambiae *M and S forms coexisted during the rainy season with a predominance of the M form in September and the peak of density being observed in August for the S form. Similar parity rates were observed in *An. arabiensis *[70.9%] (n = 86), *An*. *gambiae *M form [68.7%] (n = 64) and *An*. *gambiae *S form [81.1%] (n = 156). The circumsporozoite protein (CSP) rates were 2.82% (n = 177), 3.17% (n = 315) and 3.45% (n = 405), with the mean anthropophilic rates being 71.4% (n = 14), 86.3% (n = 22) and 91.6% (n = 24) respectively for *An*. *arabiensis *and *An*. *gambiae *M and S forms. No significant difference was observed either in host preference or in *Plasmodium falciparum *infection rates between sympatric M and S populations.

**Conclusion:**

No difference was observed either in host preference or in *Plasmodium falciparum *infection rates between sympatric M and S populations, but they present different dynamics of population. These variations are probably attributable to different breeding conditions.

## Background

The *Anopheles gambiae *complex consists of at least seven species among which *Anopheles gambiae *s.s. is one of the most anthropophilic malaria vectors in Africa [1]. The adaptation of *An. gambiae *to humans and its environment involves an ongoing speciation process that can be best demonstrated by the existence of a number of incipient taxonomic units, characterized by the presence of paracentric inversions leading to different chromosomal arrangements [2]. This speciation process is primarily observed in West Africa, where five chromosomal forms of *An. gambiae *s.s. have been described and designated with a non-Linnean nomenclature: *bamako*, *bissau*, *forest*, *mopti *and *savanna *[3,4].

During the last few years, several research teams have settled on a molecular approach to address speciation in *An. gambiae *s.s.. Various degrees of gene flow restriction were demonstrated between chromosomal forms, with strong hybrid heterokaryotype deficits in the areas of sympatry. Analysis of the rDNA intergenic spacers, located on the X-chromosome, revealed fixed sequence differences between sympatric and synchronous *savanna*/*bamako *and *mopti *populations in Mali, Burkina Faso and Cameroon [2,5,6]. To provide more insight into their taxonomic status, recent efforts have focused on the pattern of variation observed with molecular markers. This revealed the existence of two genetic variants referred to as the molecular M and S forms [7,8].

However, whatever the geographical region, it has been clearly demonstrated that the gene flow between M and S forms is very limited, revealing a current speciation phenomenon. The genetic characteristics of these forms and their known geographical distribution have recently been reviewed [9]. Studies carried out so far have shown that the M and S forms may have different habitat even in sympatric areas [5,10].

The aim of this study was to compare the dynamics of transmission of *Anopheles arabiensis *and *An. gambiae *M and S molecular forms in a Senegalese village, where the two forms coexist.

## Methods

### Study area

The village of Dielmo (13°45N, 16°25W) is located in an area of Sudan-type savanna, 280 km Southeast of Dakar and about 15 km north of the Gambian border. About 300 inhabitants are living in the village. Rainfall occurs during a four-month period, from June to October. The average annual rainfall during our study period in 2004 was 642.4 mm. Dielmo is situated on the marshy bank of a small permanent stream, the Nema, where anopheline larval sites are present all year round. Only few cattle are living in this area [11].

### Mosquito collections

Adult mosquitoes were collected monthly from July to December 2004. Two sampling methods were used: night landing catches (NLC) and pyrethrum spray catches (PSC). Hourly NLC were made on adult volunteers from 19.00 to 07.00 hours at the same two sites for three consecutive nights. Two collectors, one indoors and one outdoors, were positioned at each site. A total of twelve person-night of capture was done every month during the monthly collections. Six pyrethrum spray catches (PSC) was done during July to September early in the morning inside a total of twelve bedrooms, belonging to both types of houses, in different houses from those used for NLC.

### Field processing

Anophelines were identified to species level using morphological characteristics according to the identification keys of Gillies & De Meillon [12] and Gillies & Coetzee [13]. Ovaries from a portion of females of *An. gambiae *s.l. captured on NLC were dissected to determine parity, by observing the degree of coiling of ovarian tracheoles [14]. A total of 50–60 were randomly selected monthly. All mosquitoes belonging to the *An. gambiae *complex, dissected or not, were stored in individual tubes with silicagel and preserved at -20°C in the laboratory.

### Laboratory processing

Bloodmeal sources of a sample of the females captured by PSC were tested by enzyme-linked immunosorbent assay (ELISA) to identify bovine, ovine, caprine (sheep and goat), equine (horse and donkey), or chicken hosts [15]. Malaria infections were determined on the crushed head and thorax of all anopheline specimens by ELISA using monoclonal antibodies against *Plasmodium falciparum *circumsporozoite protein (CSP), as described by Wirtz *et al *[16]: CSP rates and 95% confidence intervals were calculated. Species identification of the *An. gambiae *complex was done by PCR [17]. Specimens identified as *An. gambiae *s.s. were tested for M and S molecular forms using the diagnostic PCR assay of Favia *et al *[2].

### Data analysis

Parity, circumsporozoite protein and anthropophilic rates were estimated. Comparisons of these proportions between M and S molecular forms and the Hardy Weinberg equilibrium were performed by X^2 ^test (P-values < 0.05 were considered to be statistically significant). The Human Biting Rate (HBR) was expressed as the average number of mosquito to bites per person per night during each month. The Entomological Inoculation Rate (EIR) was calculated by multiplying the (HBR) of each *An*. *arabiensis *and *An*. *gambiae *M and S molecular forms by the respective CSP rate, for each month.

## Results

### Species diversity

A total of 1,109 *An*. *gambiae *s.l. were caught during 72 collection nights between July and December 2004 (889 by NLC and 220 by PSC). PCR for species identification was performed on 920 (83%) mosquitoes belonging to the gambiae complex, resulting in 177 *An. arabiensis*, 316 *An. gambiae *molecular form M, 405 *An. gambiae *molecular form S and 22 hybrids M/S (Table 1).

**Table 1:** Identification of the members of *An. gambiae *complex in Dielmo

Month	*An. arabiensis*		*An. gambiae *M Form		*An. gambiae S* Form		Hybrids M/S	
	NLC	PSC	NLC	PSC	NLC	PSC	NLC	PSC
July	25	2	3	2	40	9	3	2
August	52	4	53	8	198	13	11	0
September	41	8	212	13	85	6	5	1
October	12	-	22	-	34	-	-	-
November	13	-	2	-	14	-	-	-
December	20	-	1	-	6	-	-	-

**Total**	163	14	293	23	377	28	19	3

NLC = Night landing catches

PSC = Pyrethrum spray catches.

### Parity and anthropophily rate

The mean parity rate was: 70.9% (n = 86), [CI: 60.1–80.2] for *An. arabiensis*, 68.7% (n = 64), [CI: 55.9–79.7] and 80.1% (n = 156), [CI: 73.0–86.1] for *An. gambiae *M and S forms, respectively. There was no significant difference between parity rates (X^2 ^= 4.3; p = 0.11). The proportion of mosquitoes fed on human was 71.4% (n = 14) [CI: 41.9–91.6], 86.3% (n = 22) [CI: 65.1–97.1] and 91.6% (n = 24) [CI: 73.0–98.9], respectively, for *An*. *arabiensis*, *An. gambiae *M form and S form.

### Nocturnal biting cycle

**Figure 1:**
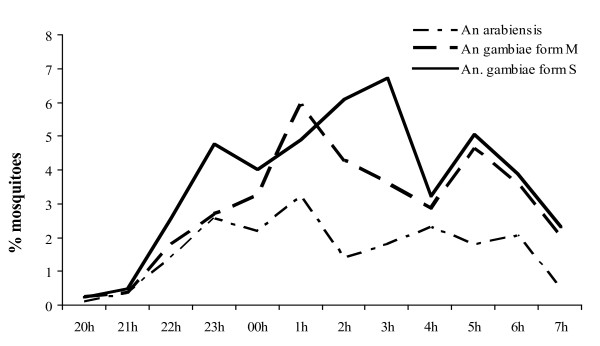
The nocturnal biting cycle of *An. arabiensis *and *An. gambiae *M and S molecular forms, from July to December 2004, in Dielmo, Senegal.

Details on hourly nocturnal biting cycle of the different species of *An*. *gambiae *s.l. are plotted in figure 1. HBR showed a consistent increased during night time reaching peaks between 11 p.m. and 5 a.m. depending on the species (Figure 1).

### Human biting rate

**Figure 2:**
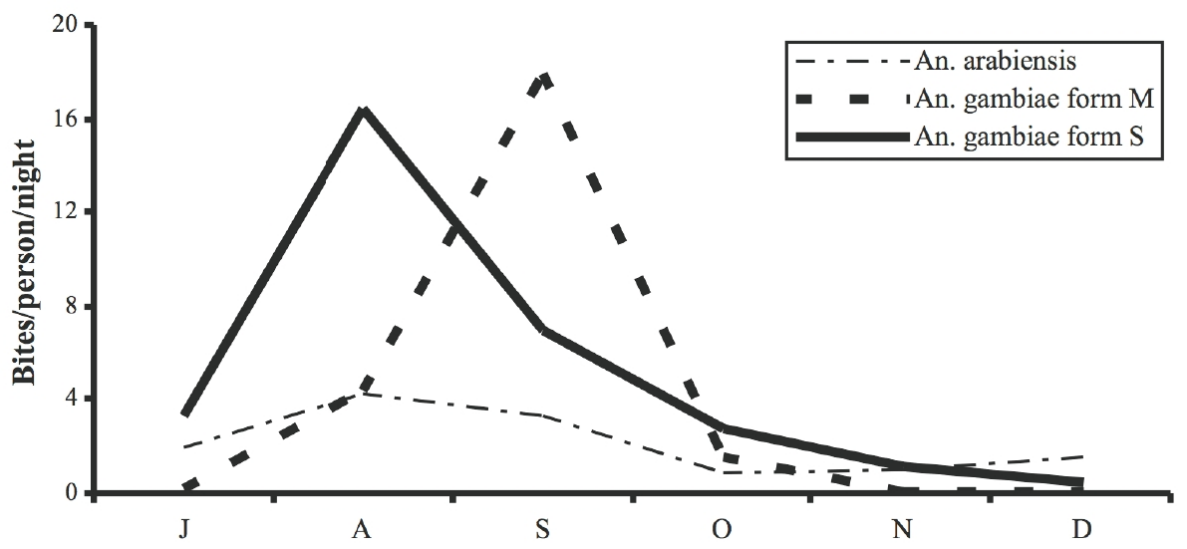
Monthly human biting rates of *An. arabiensis *and *An. gambiae *M and S molecular forms, from July to December 2004, in Dielmo, Senegal.

*An. gambiae *S molecular form biting density was maximum in August (16.5 BPN), while the M molecular form was most abundant in September (17.6 BPN). *An arabiensis*, was less abundant and the maximum was recorded in August with 4.6 BPN (Figure 2).

### Circumsporozoite protein and entomological inoculation rate

The results of CSP-ELISA assays are shown in Table 2. The mean CSP rate varied from 2.82% to 3.45%, according to the species. The differences between infection rates were not signicant (X^2 ^= 0.16; p = 0.92). Among the hybrid species M/S specimens, no CSP antigen was found. During this 6 months study period, the mean EIR was 70 infective bites/person. *An. arabiensis *and *An. gambiae *forms M and S were responsible for 17%, 35% and 48% of *P*. *falciparum *transmission respectively.

**Table 2:** Infection rate for *P. falciparum *calculated by circumsporozoite protein (CSP) ELISA from the head and thoraxes of *An. arabiensis*, *An. gambiae *M and S forms in Dielmo

Month	*An. arabiensis*		*An. gambiae *M Form		*An. gambiae *S Form	
	Tested	Positive	Tested	Positive	Tested	Positive
July	27	2	4	1	49	1
August	56	1	61	2	211	5
September	49	0	225	4	91	5
October	12	1	22	3	34	2
November	13	1	2	0	14	1
December	20	0	1	0	6	0

CSP rates [95% cl]	2.82% [0.9–6.5]		3.17% [1.5–5.8]		3.45% [1.9–5.7]	

### Hardy Weinberg equilibrium

In total, 22 hybrids M/S were obtained. The frequencies observed were 0.4 and 0.6, respectively for the molecular forms M and S. At the Hardy Weinberg equilibrium, the number of M/S hybrids expected was 366 M/S heterozygous. Difference highly significant (p < 0.0001). The number of M/S hybrids obtained was signicantly inferior in the case of panmictic crossbreeding.

## Discussion

Anopheline densities varied during this study period. This strong variation is classical in savannah areas and is related to the presence of temporary ponds during the rainy season [16]. For *An. arabiensis *this phenomenon was well known in Dielmo [1,18]. The maximum biting rate by *An. arabiensis*, *An. gambiae *M and S forms occurred after 10 p.m., when most people are in bed, as generally observed throughout Africa [19]. The late biting behaviour of the more dangerous mosquitoes is a significant finding with respect to the increase use of insecticide-treated mosquitoes nets (ITN) for the reduction of malaria. The overall parity rate is comparable to that reported by Lemasson *et al *[20] in Senegal. This indicates that the population is long lived and has the capacity to be an efficient vector of malaria during a relatively long period. The high parity rate can partially explain the CSP rates of *An. arabiensis *and *An. gambiae *in the present study which are greater than those previously recorded in other parts of Senegal [1,18,20,21]. The high CSP rate in *An*. *arabiensis *is probably explained by the high antropophilic behaviour in this particular setting. Highton *et al *[22] and Joshi *et al *[23] in the same region Kisumu, found high and similar CSP rate for *An*. *gambiae *s.s. (5.3% and 7.5% respectively), while the CSP rate was very different for *An*. *arabiensis *(0.3% and 7.5% respectively). The difference observed in *An*.*arabiensis *was due to cattle abundance and movements.

A recent analysis of published and unpublished data on the molecular forms of *An. gambiae *has demonstrated that the M form shows a more latitudinal range in West Africa than the S form, being the only form recorded in the Sahelien region of northern Senegal [9,24]. In Dielmo, the M form was mainly observed in rainy season from August to October, with a maximum in September. The S form was observed in the rainy season as well, with a maximum in August, but was also found during the dry season. *An*. *arabiensis *had an approximately constant density during the rainy season and the beginning of the dry season, without an obvious peak. Thus rainfall alone can not explain fluctuations observed in M and S forms densities.

Cytogenetic studies were conducted in 1991 in Dielmo and revealed an important chromosomic polymorphism. Thirty-two half-gravid females collected in January and February 1991 showed chromosomal inversions characteristic of *bissau *chromosomal form which belongs to the M molecular form [8]. Twenty-four females collected in September 1991 mainly revealed *savanna *cytotype which belongs either to M form or S form [8]. In this study, the replacement of S molecular form by M molecular form in the late rainy season might reflect the replacement of *savanna *chromosomal form by *bissau *chromosomal form. Climate (rainfall, temperature, relative humidity) as well as nature of anopheline larval development sites are dramatically change throughout the year during the rainy season. As chromosomal inversions are known to be involved in adaptation to climate and environment [1], the correspondence of molecular and chromosomal forms may explain the distribution pattern of molecular forms.

In West Africa, there is evidence of varying levels of hybridation between M and S forms, a mechanism by which adaptive genes may flow from one to the other, including those conferring insecticide resistance [25,26]. No, or very few, M/S hybrids were observed throughout Cameroon [6,27], Ghana [10], Mali and Burkina Faso [9]. In this study, 22 M/S hybrids were observed (3.05%). Despite this number of hybrids, the *An. gambiae *population is far from Hardy Weinberg equilibrium suggesting restricted gene flow between M and S form. The occurrence of M/S hybrids in the field, however, has been reported several times. Della Torre *et al *[8] found three hybrids patterns out of 1,161 *An. gambiae *adult mosquitoes tested from throughout Africa (hybrid frequency = 0.26%). The authors, therefore, mentioned contamination as a possible cause of the hybrid patterns they reported. Further evidence for the viability of M/S hybrids in the wild was provided by Taylor *et al *[28], who reported occurrence of M/S hybrid larvae at a frequency of 0–1.29% in Banambani (Mali). Tripet *et al *[29] identified an inseminated female showing the M/S pattern, which demonstrated that M/S hybrids could be produced in the field, survive up to the adult stage and are reproductively active.

## Conclusion

*Anopheles arabiensis *and the M and S molecular forms of *An. gambiae *coexist in Dielmo village. No difference was observed either in host preference or in *Plasmodium falciparum *infection rates between sympatric M and S populations, but they present different dynamics of transmission: the S form is the major vector during the first part of the rainy season and is replaced by M form later in the season. These variations are probably attributable to different breeding conditions.

## Authors' contributions

MON and DF have equally contributed to the design, acquisition, analysis, interpretation of data and manuscript drafting. LK contributed to conception of study and contributed markedly to the analysis of entomological data. CB for field activities and Molecular biology. JFT and CB participated in the conception and coordination of the study and helped to draft the manuscript. CS provided the scientific supervision in Dielmo.
